# Recent Progress in Polyion Complex Nanoparticles with Enhanced Stability for Drug Delivery

**DOI:** 10.3390/polym16131871

**Published:** 2024-06-30

**Authors:** Xinlin Ma, Tianyi Zhao, Xiaoyue Ren, Hui Lin, Pan He

**Affiliations:** 1 School of Chemistry and Environmental Engineering, Changchun University of Science and Technology, Changchun 130022, China; 2 College of Pharmacy, Changchun University of Chinese Medicine, Changchun 130117, China; 3 School of Materials Science and Engineering, Changchun University of Science and Technology, Changchun 130022, China

**Keywords:** polymeric micelles, electrostatic interactions, improved stability, protein delivery, therapeutic drug carriers, polyion complex vesicles

## Abstract

Polyion complex (PIC) nanoparticles, including PIC micelles and PICsomes, are typically composed of poly(ethylene glycol) block copolymers coupled with oppositely charged polyelectrolytes or therapeutic agents via electrostatic interaction. Due to a simple and rapid preparation process with high drug-loading efficiency, PIC nanoparticles are beneficial to maintaining the chemical integrity and high biological activity of the loaded drugs. However, the stability of PIC nanoparticles can be disrupted in high-ionic-strength solutions because electrostatic interaction is the DRIVING force; these disruptions can thus impair drug delivery. Herein, we summarize the advances in the use of PIC nanoparticles for delivery of charged drugs, focusing on the different chemical and physical strategies employed to enhance their stability, including enhancing the charge density, crosslinking, increasing hydrophobic interactions, forming hydrogen bonds, and the development of PIC-based gels. In particular, we describe the use of PIC nanoparticles to load peptide antibiotics targeting antibiotic-resistant and biofilm-related diseases and the use of nanoparticles that load chemotherapeutics and gaseous donors for cancer treatment. Furthermore, the application of PIC nanoparticles as magnetic resonance imaging contrast agents is summarized for the first time. Therefore, this review is of great significance for advances in the use of polymeric nanoparticles for functional drug delivery.

## 1. Introduction

Polymers with positive or negative charges are called polyelectrolytes [1]. Polyelectrolyte complexes (PECs) are formed by the electrostatic interaction of oppositely-charged polyelectrolytes in aqueous solution [2]. Nanoscale PEC micelles are commonly referred to as polyion complex (PIC) micelles, interpolyelectrolyte complex micelles, block ionomer complex micelles, or complex coacervate core micelles [3]. Typically, PECs are unstable and undergo flocculation, leading to coacervation. Therefore, most PIC micelles include a layer of hydrophilic poly(ethylene oxide) (PEO) or poly(ethylene glycol) (PEG) polymers as the shell, which can improve their colloidal stability [4]. In addition, the inclusion of PEG can effectively avoid the nonspecific adsorption of proteins and can prevent macrophage uptake in vivo, making the nanosized micelles “stealthy” to some extent and thus prolonging their circulation time in the blood [5]. Depending on the ratio of hydrophilic PEG chain length to the length of the hydrophobic chains, various self-assembled nanostructures have been observed, such as core-shell spherical micelles, cylindrical micelles, and enclosed membrane structures (vesicles, also known as polymersomes) [6]. In this review, the term “PIC nanoparticles” refers to spherical and cylindrical PIC micelles and to polyion complex vesicles (PICsomes).

The history of PIC nanoparticles can be traced back to 1995. Harada and Kataoka combined two block copolymers with opposite charges in aqueous solution and obtained PIC micelles with a narrow size distribution [7]. In particular, the first PIC micelle consisted of poly(ethylene glycol)-poly(L-lysine) and poly(ethylene glycol)-poly(α,β-aspartic acid) (PEG-P(Asp)). These PIC micelles are spherical particles without any secondary aggregates and with a hydrodynamic radius at infinite dilution measured to be 15.2 nm. Since then, Kataoka’s group has constructed a series of novel PIC nanoparticles for drug delivery, especially for delivery of plasmid DNA and messenger RNA [8]. Unlike classic polymeric nanoparticles formed through hydrophobic interactions, PIC nanoparticles have total solubility in water and a very mild and rapid preparation process that does not involve any harmful solvents, which make them safe and excellent vehicles for fragile therapeutics such as proteins and other naturally occurring polyelectrolytes.

**Figure 1:**
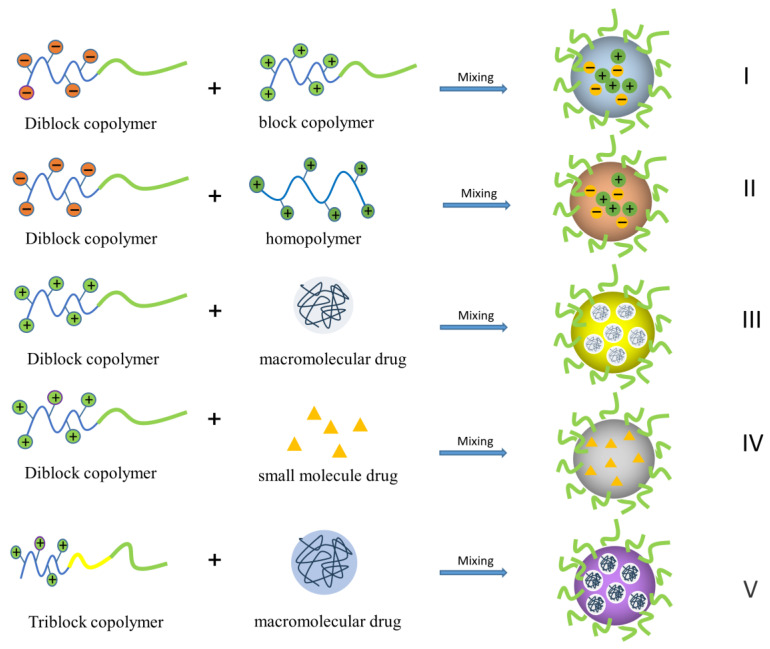
Schematic illustration of different types of PIC micelles discussed in this review. The macromolecular drugs and small-molecule drugs are shown to be negatively charged in this figure.

To date, PIC nanoparticles have been used as efficient drug vehicles for protein drugs [9], gene-based therapies [10], anticancer drugs, and heparin [11], as well as for basic and clinical research. As shown in Figure 1, two-component PIC micelles can be divided into the following types: (I) diblock copolymers with different charges; (II) diblock copolymers and homopolymers; (III) diblock copolymer carriers of different charged macromolecule drugs such as nucleic acids, proteins, and drugs; (IV) diblock copolymer carriers of small-molecule drugs such as anticancer drugs and gaseous donor drugs; and (V) triblock copolymer carriers of macromolecule drugs. Compared to other drug carriers such as polymeric micro/nanoparticles fabricated by emulsion techniques, nanogels fabricated by crosslinking, non-polymeric liposomes, and solid lipid nanoparticles, PIC micelles fabricated by simple mixing of two oppositely charged species perform best in maintaining the chemical integrity and high biological activity of the loaded drugs, with higher drug-loading capacity and encapsulation efficiency [12]. Furthermore, the PEG shell of PIC can prolong the shelf life and in vivo stability of loaded drugs like peptides, proteins, and oligonucleotides. However, since PIC nanoparticles are mainly driven by Coulomb forces, their stability is usually poor and is influenced by the ionic strength and pH of their environment. To this end, various chemical and physical strategies have been developed to improve the stability of PIC nanoparticles for better pharmacokinetics and enhanced drug delivery.

**Figure 2:**
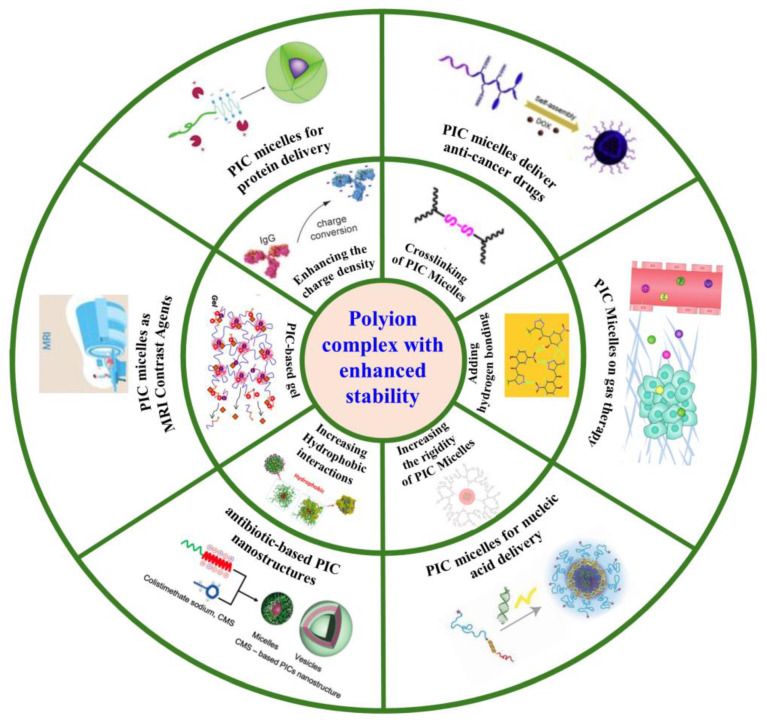
Schematic illustration of the main strategies used for improving the stability of PIC nanoparticles and their drug-delivery applications.

This review first mainly focuses on the strategies developed to improve the stability of PIC nanoparticles over the past decade, including enhancing the charge density, crosslinking, increasing hydrophobic interactions, adding hydrogen bonds, forming a PIC-based gel, and increasing the rigidity of the PIC-nanoparticle component. Furthermore, we discuss in detail the application of stability-enhancing PIC nanoparticles for the delivery of protein drugs, antibiotics, gaseous donors, and genetic medicines and their application as a magnetic resonance imaging (MRI) contrast agent (Figure 2).

## 2. Strategies to Improve PIC Stability for Protein Delivery

**Figure 3:**
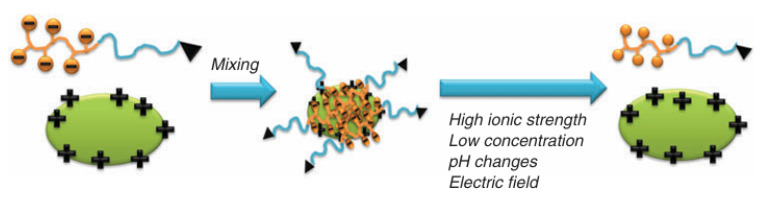
Formation of polyion complex micelles and their disintegration under conditions of high ionic strength, dilution, pH changes, and the presence of an electric field in the environment. Image reproduced from the reference [4].

The activity of protein drugs in vivo is affected by factors such as surface charge, large size, weak tertiary structure, and short half-life [4]. Delivery of protein drugs by PIC nanoparticles can help protect their therapeutic activity and prolong their circulation time. However, as shown in Figure 3, high ionic strength, dilution, and pH changes in the medium may alter the electric field surrounding the polymer, resulting in cracking of the PIC micelle. In this section, we summarize methods developed to improve the stability of PIC micelles for protein delivery, including enhancing the charge density of each component to produce stronger electrostatic interactions, crosslinking of the micelles, increasing hydrophobic interactions, introducing hydrogen bonding, and other methods.

### 2.1. Improving the Charge Density of PIC Components

Increasing the electrostatic interaction by increasing the charge density can greatly improve the stability of PIC micelles [13]. Different components of PIC nanoparticles have been modified for this purpose, such as enhancing the charge density of protein drugs, increasing the length of the charged segment of the block copolymer, and incorporating the charged homopolymer as the third component. 

#### 2.1.1. Enhancing the Charge Density of the Protein Drugs

**Figure 4:**
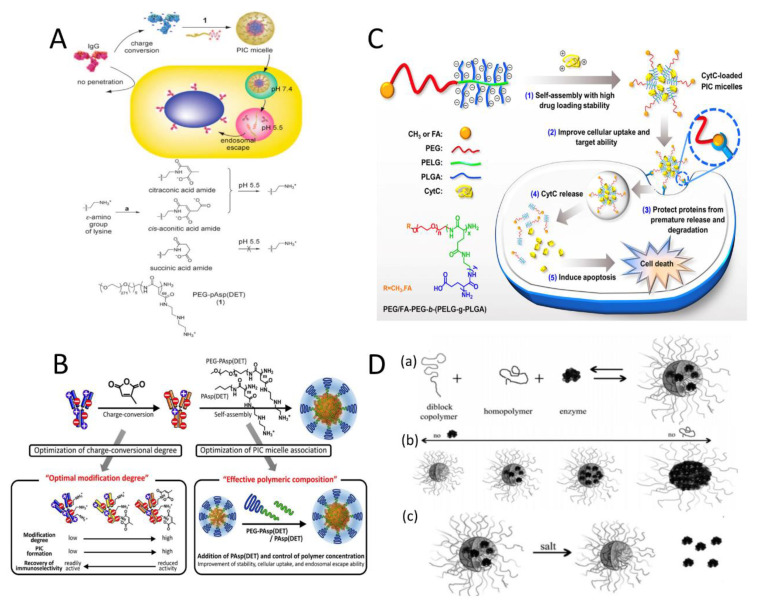
(**A**) Formation of charge-conversional PIC micelles between PEG-pAsp(DET) and IgG derivatives modified by citraconic anhydride, cis-aconitic anhydride, or succinic anhydride [14]. (**B**) Three-component PIC micelles containing charge-converted IgG-antibody derivatives [15]. (**C**) Preparation of PIC micelles loaded with CytC and its intracellular transport pathway [16]. Reproduced from [16] with permission from the SpringerLink. (**D**) Schematic depicting (a) the preparation of PIC micelles upon mixing of diblock copolymer, enzyme, and homopolymer; (b) effects of different proportions of enzymes and homopolymer on micelle morphology, and (c) PIC micelles release enzymes when exposed to external salt-jump stimulus [17].

Lee et al. [14] modified the ε-amines of lysine residues of proteins into charged conversional moieties, citraconic acid amide (Cit), or cis-aconitic acid amide (Aco). The negative charge density of modified proteins can be significantly increased to form stable PIC micelles with cationic block copolymers even at physiological salt concentrations. In this way, PEG-poly{N-[N-(2-aminoethyl)-2-aminoethyl] aspartamide} (PEG-pAsp(DET)) and different immunoglobulin G (IgG) molecules have been used to develop novel charge-conversional PIC micelles. After cellular uptake, the Cit and Aco groups rapidly degraded to produce lysine at the endosomal pH of 5.5, resulting in regeneration of the original proteins and the dissociation of the PIC micelles. Meanwhile, the release of a free cationic block copolymer induced pH-dependent instability of the endosomal membrane and helped the endosomal escape of the protein into cytoplasm of living cells (Figure 4A).

Similarly, in 2015, Kim et al. obtained IgG derivatives with appropriate charge conversion using Cit. Three-component PIC micelles were prepared combining IgG with PEG-PAsp(DET) and the homopolymer poly{N-[N-(2-aminoethyl) -2-aminoethyl]aspartamide}(PAsp(DET)), in which the homopolymer enhanced the electrostatic interaction (Figure 4B). The study revealed that a PIC micelle, modified with an IgG derivative featuring 50% Cit conversion and 75% PAsp (DET), in a polymer/antibody molar ratio of 4:1, significantly improved the intracellular delivery of antibodies [15]. 

#### 2.1.2. Increasing the Charge Density of the Charged Segment of the Block Copolymers

In addition to protein-based drugs, enhancement of the charge density of carrier polymers can also improve the stability of PIC micelles. In 2020, Li et al. developed a series of folate (FA)-modified PEG-based copolymers, specifically, FA-functionalized PEG-*b*-(poly(2-aminoethyl-L-glutamate)-*g*-poly(L-glutamic acid))_s_ (FA-PEG-*b*-(PELG-*g*-PLGA))_s_, as potential carriers for Cytochrome C (CytC) [16]. The charge density and graft density of the copolymer was designed by adjusting the length of the PLGA brush. As shown in Figure 4C, stable PIC micelles were formed by combining negatively charged FA-PEG-*b*-(PELG-*g*-PLGA)_s_ with positively charged CytC. Notably, compared with copolymers consisting of PEG and linear charged hydrophilic blocks, the block copolymers with brush-like PLGA segments demonstrated enhanced drug-loading stability, growth suppression, and apoptotic activity. This might be attributed to the improved electrostatic interactions between brushed charged polymers and loaded CytC, thus increasing the drug-loading stability and protecting encapsulated proteins from degradation.

#### 2.1.3. Incorporation of Charged Homopolymers

In a high-ionic-strength environment, PIC micelles can be stabilized and prevented from disassembling by adding a homopolymer that carries a charge similar to that of the system. The principle is that the homopolymer has a higher charge density than the protein globules, resulting in a stronger electrostatic interaction .As shown in Figure 4D(a), adding homopolymer poly(N,N-dimethylaminoethyl methacrylate) (PDMAEMA_150_) to the two-component micelle system of poly(acrylic acid)-block-poly(acryl amide) (PAA_42_-*b*-PAAm_417_) and the lysozyme contributed to the micelle stability by increasing resistance to ionic solutions [17]. The fraction of homopolymer in the core increased and the shape of the micelle cores gradually changed from ellipsoidal to sphere. Interestingly, by changing the proportion of homopolymers in the system, the load of proteins in the core can be changed Figure 4D(b). It is generally recommended to use excessive homopolymer to form stable PIC micelles, such as lysozyme/homopolymer 7:13. More interestingly, the PIC micelle of the three-component system can maintain its structural integrity after protein release Figure 4D(c), whereas the conventional two-component PIC micelles eventually disassembled.

### 2.2. Introducing the Crosslinked Structure in PIC Micelles 

**Figure 5:**
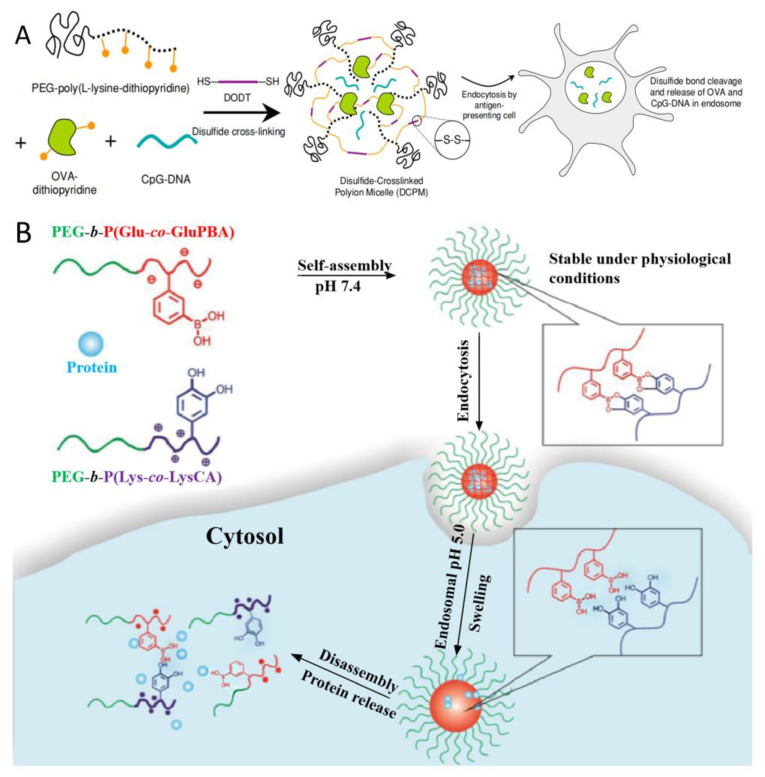
(**A**) Formation of disulfide-crosslinked PIC micelles, their uptake by antigen-presenting cells, and the dissociation by the reduction of disulfide bonds within cells [18]. Reproduced from [18] with permission from the Springer Nature. (**B**) Illustration of boronate-ester-linked PIC micelles and their intracellular protein delivery triggered by endosomal pH [19]. Reproduced from [19] with permission from American Chemical Society.

Crosslinking is a feasible method for improving the stability of PIC nanoparticles. In 2009, disulfide crosslinking was employed in PIC, producing a stable but degradable protein-delivery system. As shown in Figure 5A, disulfide-crosslinked PIC micelles (DCPM) were prepared with PEG-poly(L-lysine-dithiopyridine), ovalbumin (OVA), immunostimulatory CpG-DNA and 3,6-dioxa-1,8-octanedithiol. After they have been internalized by antigen-presenting cells, DCPM can release CpG-DNA and OVA in the endosomes due to the cleavage of disulfide bonds by intracellular GSH [18].

The phenylboronic acid-catechol interaction was also applied in PIC micelles with crosslinked structures. Ren et al. [19]. developed pH-/sugar-sensitive PIC micelles based on PEG-*b*-P(Glu-*co*-GluPBA) and PEG-*b*-P-(Lys-*co*-LysCA) for intracellular protein delivery (Figure 5B). The PIC micelles core-cross-linked by boronate ester bonds exhibited superior physiological stability under physiological conditions but disassemble and thus release the loaded proteins in the presence of excess fructose or at endosomal pH.

In 2020, Kataoka et al. [20] developed glucose oxidase (GOD)-loaded PICsomes composed of anionic PEG-PAsp and poly([2-[[1-[(2-aminoethyl)thio]-1-methylethyl] thio]ethyl]-α,β-aspartamide) (PATK) as polycation segments. 1-ethyl-3-(3-dimethylaminopropyl) carbodiimide (EDC) was applied for covalent cross-linking of the membrane network. When exposed to H_2_O_2_, the cleavage of reactive oxygen species (ROS)-responsive thioketal linkers in PATK leads to PIC expansion without fracture, allowing the release of the loaded glucose oxidase and causing cancer cells to die from glucose starvation.

### 2.3. PIC Reinforced by Hydrophobic Interactions 

**Figure 6:**
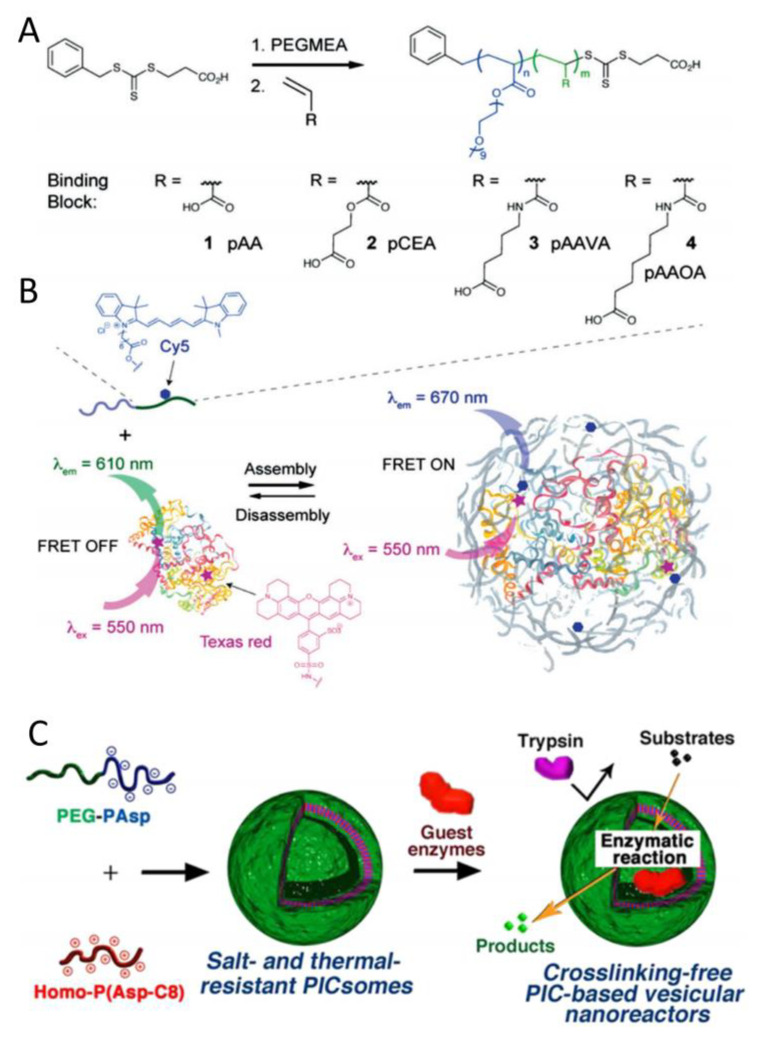
(**A**) The route for the synthesis of polymers with different lengths of carbon spacers by RAFT polymerization. (**B**) PIC micelles were prepared with the Cy5-labeled polymer and Texas red-labeled lysozyme [22]. Reproduced from [22] with permission from Wiley VCH. (**C**) Formation of C8-PICsomes with Improved Physiological Stability as Enzymatic Nanoreactors [24].

Beyond electrostatic attractions, the incorporation of hydrophobic interactions improves the stability of PIC nanoparticles, particularly in the presence of high salt concentrations [21]. Li et al. synthesized block copolymers with four different pendant groups by RAFT polymerization (Figure 6A), including acrylic acid (AA) (1), carboxyethyl acrylate (CEA) (2), acryloyl aminovaleric acid (AAVA) (3), and acryloylaminooctanoic acid (AAOA) (4) [22]. Increasing the length of the carbon spacers between -COOH and the polymer backbone led to an increase in p*K*_a_. Additionally, the corresponding PIC micelles formed with a cationic lysozyme had significantly higher stability at physiological pH and improved protein-release behavior after cell encapsulation. Specifically, by labeling the polymer with Sulfo-Cyanine5 (Cy5) and the protein with Texas red, polymer assembly was studied using the Fluorescent Resonance Energy Transfer (FRET) technique (Figure 6B). Later, the researchers increased the number of carbon spacers to five or eight and found that the binding strength of AAVA and AAOA spacers was two to three times greater than that of AA and CEA due to the additional interaction between the protein and the hydrophobic pockets on the polymer, which drove water away from the protein during the micelles’ self-assembly [23]. The penetration of micelles into Michigan Cancer Foundation-7 (MCF-7) breast-cancer-cell spheroids was studied by light-sheet fluorescence microscopy. Compared with the representative “PIC-like” micelle, stable micelles with carbon spacers could deliver lysozymes into MCF-7 breast-cancer-cell spheroids more efficiently.

Similarly, enhanced stability of PICsomes was also found to result from increasing the hydrophobicity of polyelectrolytes with longer aliphatic side chains. In 2014, Chuanoi et al. prepared the first stable non-cross-linked PICsomes under physiological conditions by using PEG−PAsp and homopolymer poly(aspartamide) with varying lengths of alkyl spacers (Homo(PAsp-Cx: No. x=5 or x=8)) [24]. It was demonstrated that PICsomes generated from Homo-P(Asp-C8) (C8-PICsome) could maintain the vesicular structures in the presence of 150 mM NaCl, even at elevated temperatures. As shown in Figure 6C, a C8-PICsome with octyl side chains was able to encapsulate enzymes without disrupting the original enzymatic activity and to act as a nanoreactor even in the presence of protease.

### 2.4. PIC Reinforced by Hydrogen Bonds

**Figure 7:**
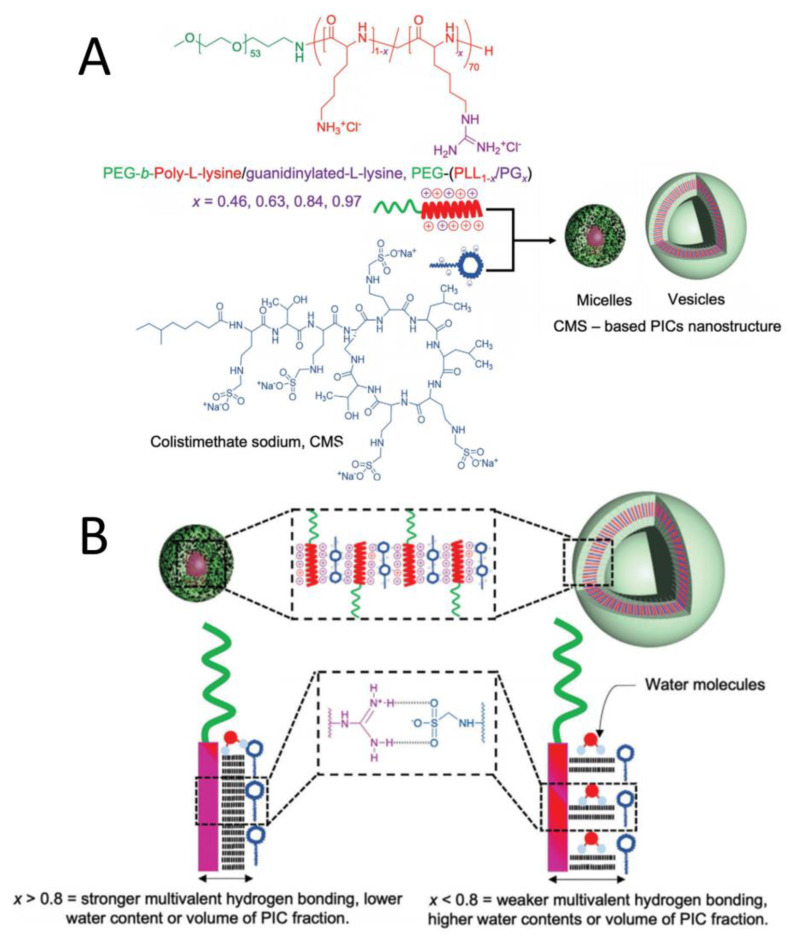
(**A**) Illustration of PIC preparation from colistimethate sodium and a range of block copolymers with side chains modified by a guanidine group. (**B**) Different PIC nanostructures are based on the guanidine-modification ratio (the dashed line shows hydrogen bonds in the diagram) [26]. Reproduced from [26] with permission from Wiley VCH.

In addition to protein drugs, polypeptide-based antibiotics can also be delivered by PIC nanostructures as a strategy to overcome antibiotic resistance. As illustrated in Figure 7A, a guanidine group was introduced to side chains to form different block copolymers, called polyethylene glycol (PEG)-poly-L-lysine/guanidinylated-L-lysine(PEG-(PLL_1−x_/PG_x_)), where x refers to the guanidine-modification ratio. PIC was formed from these polymers and antibiotic colistimethate sodium (CMS) [25]. The guanidine groups enhanced the interaction between cations and CMS by forming multivalent hydrogen bonds. Furthermore, micellar and vesicular PIC nanostructures were selectively formed based on the ratio of guanidine residues. When the x value was <0.8, fewer multivalent hydrogen bonds were formed, resulting in more water molecules being deposited in the PIC domain, which increased the volume of PIC and promoted the formation of vesicles. When the x value was >0.8, more multivalent hydrogen bonds were formed, increasing the hydrophobicity and promoting the formation of PIC micelles (Figure 7B) [26]. Furthermore, using glutaraldehyde (GA) crosslinking, CMS release and antimicrobial activity can be modulated, particularly in PIC vesicle formulations. Thus, CMS-based PICs are promising for overcoming antibiotic resistant and biofilm-related diseases. 

Fay et al. [27] developed two novel polymer systems: poly(sarcosine) _127_-*b*-poly(glutamic acid)_50_ and poly(methyl-2-oxazolines)_38_-*b*-poly(oxazolepropanoic acid)_27_-*b*-poly(methyl-2-oxazoline)_38_. These polymers interacted with brain-derived neurotrophic factor (BDNF) through electrostatic attractions and hydrogen bonding, leading to the formation of a novel PIC with the potential for therapeutic activity in neurological diseases.

### 2.5. Other Methods to Enhance Stability of PIC

**Figure 8:**
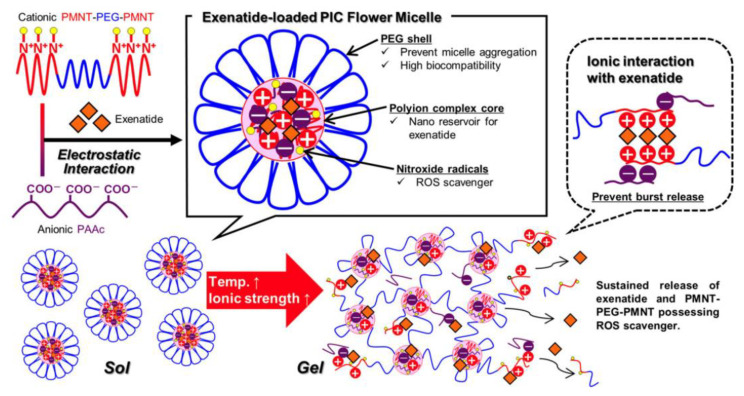
Schematic illustration of exenatide-loaded, redox-active, injectable gel based on PIC flower micelles [28]. Reproduced from [28] with permission from Wiley VCH.

A triblock copolymer PMNT-PEG-PMNT consisting of polyamine (PMNT) and PEG was combined with poly(acrylic acid) to develop a PIC-based redox-active injectable gel (RIG) for the delivery of exenatide [28]. Interestingly, these PIC flower-micelles solutions showed an irreversible sol-gel transition with increasing temperature and ionic strength and formed gels under physiological conditions [29]. The PIC-based gel could provide a sustained release of exenatide without a significant initial burst (Figure 8). In addition, the ROS-scavenging property of PMNT-PEG-PMNT could help to suppress oxidative events and to preserve the islet structure. These findings suggest that exenatide-loaded RIG has the potential to enhance the efficacy of treatment for type 2 diabetes. Introduction of dendrimers and preparation of stable PIC micelles by increasing the rigidity of the dendritic architecture are other excellent strategies and have been summarized in another review [30]. 

## 3. PIC Nanoparticles for Anticancer Drug Delivery

PIC nanoparticles have garnered significant attention in cancer therapy due to their advantages in terms of easy preparation, efficient drug encapsulation and bio-specific responsiveness. Herein, we summarize the application of PIC micelles for cancer therapy, including delivery of chemotherapeutic drugs, photosensitizers, metal ions, and NO donors.

### 3.1. PIC Delivery of Chemotherapy Drugs

**Figure 9:**
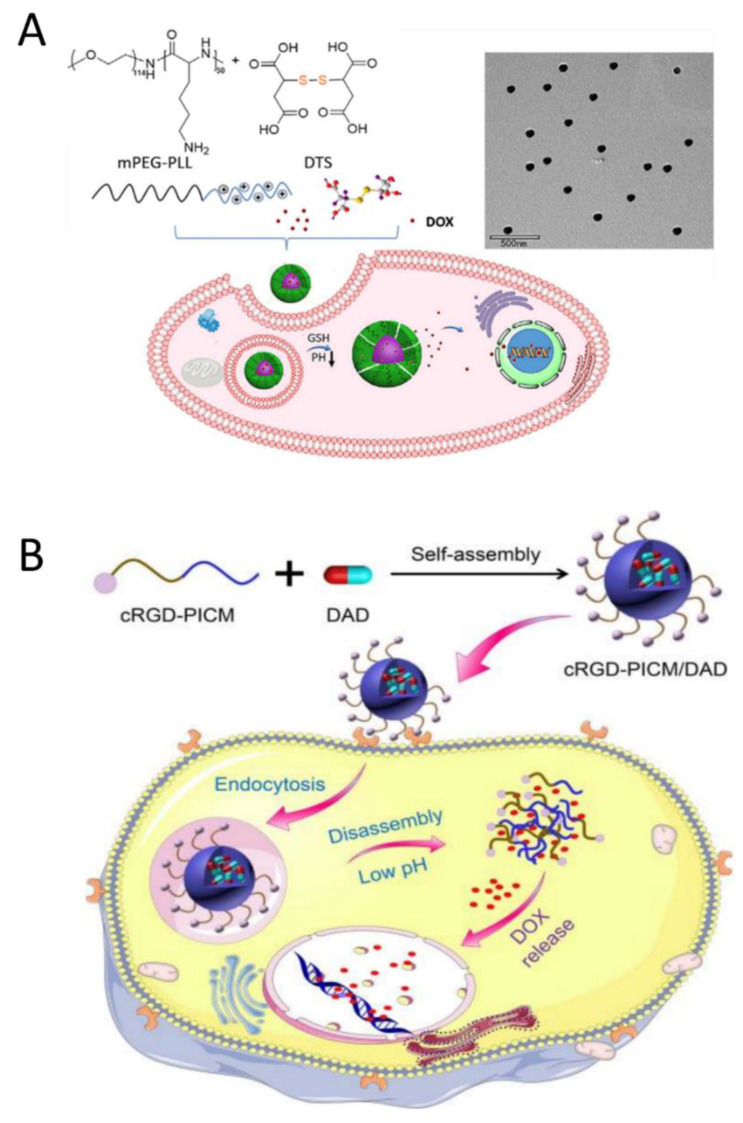
(**A**) Schematic illustration of the preparation of DOX-loaded PIC micelles for intracellular drug delivery [31]. Reproduced from [31] with permission from Elsevier. (**B**) Schematic illustration of targeted intracellular drug delivery by pH-responsive PIC micelles [32]. Reproduced from [32] with permission from Chinese Chemical Society.

In 2018, Du et al. developed a PIC micelle carrier for doxorubicin (DOX) that was responsive to both pH and reduction. The micelles were formed from poly(ethylene glycol)-poly(L-lysine) (mPEG-PLL), DOX, and dithiodisuccinic acid (DTS) in an aqueous solution (Figure 9A). The presence of four carboxyl groups in DTS ensured the stability of PIC micelles, and the disulfide bonds in DTS endowed micelles with a reduction-responsive property. These DOX-loaded micelles effectively inhibited the growth of MCF-7 human breast cancer cells [31]. Similarly, Ding et al. prepared a pH-responsive and targeted multifunctional micellar by using cRGD-modified PEG-PLL and 2,3-dimethyl maleic anhydride-modified doxorubicin (DAD) (Figure 9B). The incorporation of cRGD peptides gives micelles the ability to selectively recognize αvβ3 integrins, thereby facilitating targeted drug delivery to cancer cells. In a simulated acidic environment, the PIC micelles loaded with DAD exhibit sustained release of DOX, thus enhancing their anticancer efficacy [32]. 

By using oppositely charged PEG-P(Asp) and poly(2-hydroxyethyl methacrylate)-*b*-poly(L-lysine), Kalinova et al. designed surface-charge-adjustable micelles decorated with cell-targeting ligands for curcumin delivery. The calculated drug-loading-efficiency values were between 60% and 71% [33]. These functional PIC micelles have potential for use in cancer treatment.

### 3.2. PIC Delivery of Gaseous Donors for Cancer Therapy 

Gas therapy is a prospective cancer-treatment method due to its inherent avoidance of drug resistance and its biosafety [34]. Gas transmitters include hydrogen sulfide, nitric oxide (NO) [35,36], and carbon monoxide [37]. Kudo et al. prepared PIC micelles containing poly(L-arginine) to achieve controlled delivery of arginine (NO donor) to the targeted site for potent NO-based cancer therapy [38]. In brief, micelles were formed from PEG-*b*-poly(L-arginine) with polyanions such as hyaluronic acid (HA) or chondroitin sulfate C (CS). PICs prepared with HA were less stable than those prepared with CS, which may be due to the stronger Coulomb forces between carboxylate/sulfate in CS and guanidine in PEG-*b*-PArg. At the same time, the guanidine group in PEG-*b*-PArg not only binds to CS by Coulomb forces, but also interacts with them via hydrogen bonds, leading to successful formation of supramolecular assemblies. 

Sulfur dioxide (SO_2_) has long been considered a harmful atmospheric pollutant and has received less attention. However, SO_2_ was recently found to be produced endogenously in mammals and to have important pathophysiological functions, especially in the cardiovascular system [39]. Inspired by the potential anticancer applications of SO_2_, our group recently developed PIC micelles consisting of negatively charged PEG-*b*-poly(L-glutamic acid) and positively charged 2,4-dinitrophenylsulfonamide compounds as an SO_2_ prodrug. The in vitro anticancer evaluation of this PIC micelles is ongoing. 

### 3.3. PIC Nanoparticles Containing Other Drugs for Cancer Therapy

**Figure 10:**
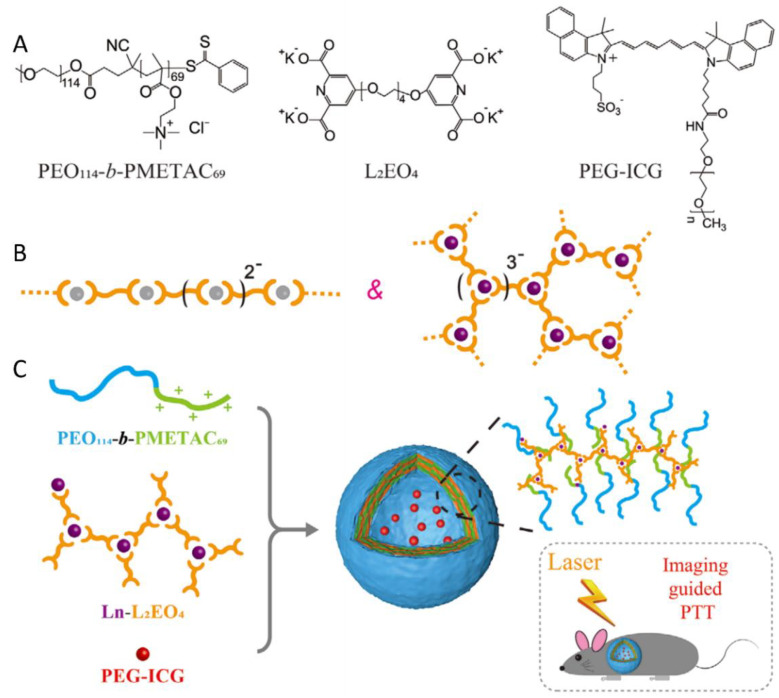
Formation of PIC vesicles based on neutral-cationic diblock copolymers, branched Ln-L_2_EO_4_ coordination polymers, and their encapsulation of PEG-ICG (red dots) for PTT application [40]. (**A**) The chemical structures; (**B**) Scheme illustration of the coordination of metal ions with two or three DPA groups; (**C**) Scheme of the three-component PIC formation and the PTT application. Reproduced from [40] with permission from Wiley VCH.

Stuart et al. prepared a novel PIC vesicle by using neutral-cationic PEO-*b*-poly(2-(methacryloyloxy)ethyl trimethylammonium chloride) (PEO_114_-*b*-PMETAC_69_) and supramolecular coordination polyelectrolyte. These micelles were formed through full coordination between a bis-ligand 1,11-bis(2,6-dicarboxypyridin-4-yloxy)-3,6,9-trioxaundecane(L_2_EO_4_) including two dipicolinic acid (DPA) groups and different metal ions [40]. PEGlated indocyanine green (PEG-ICG) was successfully encapsulated as a model photothermal transduction agent (Figure 10A). Notably, the stability of PIC vesicles is enhanced by the tunable function and branched chain structure of the Ln-L_2_EO_4_ coordination polymer. For the transition of metal ions (Zn^2+^, Mn^2+^, Co^2+^, Ni^2+^), each metal ion is completely coordinated with two DPA groups when the metal ion/DPA ratio is 1:2. In particular, lanthanide metal ions (Ln^3+^), can coordinate three DPA units at a Ln^3+^/DPA ratio of 1:3 (Figure 10B). Furthermore, gadolinium (Gd^3+^) has also been selected as the metal ion to investigate the co-assembly of PEO_114_-*b*-PMETAC_69_ with Gd-L_2_EO_4_ at pH 7.4 (Figure 10C). Overall, these vesicles showed high stability, efficient photothermal conversion, good biocompatibility, and good behavior for the thermal ablation of tumors.

Lee et al. [41] synthesized a novel block copolymer (PEG-TPE-PEI) by incorporating tetraphenylethene (TPE) groups as aggregation-induced emission-enhancement (AIE) units into the center of the polymeric segment of PEG and polyethyleneimine(PEI) and successfully prepared PIC micelles with dendrimer porphyrin (DP). Under broad-band visible-light illumination, PIC micelles showed photocytotoxicity. In particular, the TPE group emitted enhanced fluorescence and the formation of PIC micelles could be directly observed. Karayianni et al. [42] developed PIC micelles with photosensitive porphyrin. The optical properties of the PICs were directly correlated with the inherent protonation and/or aggregation state of 5,10,15,20-tetrakis-(4-sulfonatophenyl)-porphyrin, which was obviously further enhanced upon interaction with block copolymers.

## 4. PIC Nanoparticles for Delivery of Nucleic-Acid Drugs 

The use of polymeric micelles for gene therapy have been reviewed elsewhere [43]. Here, we provide an overview only of studies from the past decade that focus on increasing the stability of PIC nanoparticles and improving the efficacy of nucleic-acid therapy.

### 4.1. PIC Nanoparticles Stabilized by Crosslinking for siRNA Delivery

**Figure 11:**
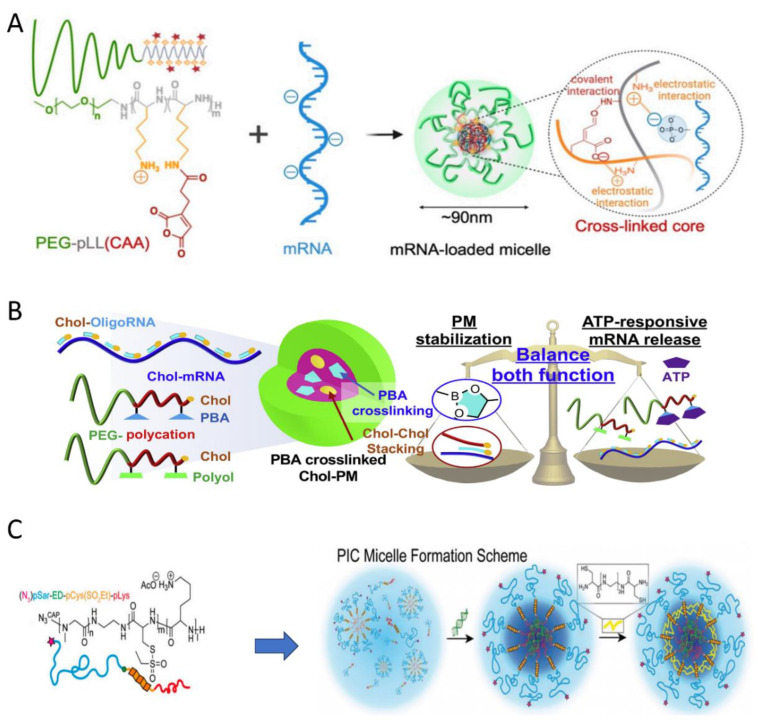
(**A**) Preparation of micelles loaded with mRNA based on covalent interactions and electrostatic interactions [44]. The asterisks represent cis-aconitic anhydride on PEG-*b*-polylysine. (**B**) Formation of ATP-responsive polyplex micelles with optimal density of phenylboronate-ester crosslinking for mRNA delivery [45]. Reproduced from [45] with permission from Elsevier. (**C**) Scheme of PIC micelle formation by interaction with oligonucleotides and subsequent crosslinking by cTETAc to introduce covalent disulfides for intrinsic stability (displayed in bright yellow) [46]. Reproduced from [46] with permission from Wiley VCH. The asterisks in subfigure A represent cis-aconitic anhydride on PEG-b-polylysine.

Messenger RNA (mRNA) is emerging as a promising therapeutic modality for a variety of diseases. Yang et al. developed mRNA-incorporated pH-responsive PIC micelles [44]. The reaction between the cis-aconitic anhydride (CAA) portion and primary amines could crosslink the core by forming pH-sensitive amide bonds, while residues of amino groups in the CAA-modified poly(L-lysine) segment facilitated ion complexation with mRNA (Figure 11A). Notably, these crosslinked micelles can effectively prevent loaded mRNA from being degraded by nuclease, thus enhancing the efficiency of mRNA delivery.

In 2021, Kataoka et al. [45]. designed mRNA-loaded polyplex micelles (PMs) responsive to adenosine triphosphate (ATP). As shown in Figure 11B, the PMs were formed by short RNA oligonucleotides possessing a Chol moiety (Chol-OligoRNAs), Chol-decorated block copolymers derivatized with phenylboronic acid, and PEG-polycations with polyol groups. The stability of these PIC micelles was enhanced both by the stacking of Chol moieties and by crosslinking structures via spontaneous phenylboronate ester formation. Additionally, the obtained PMs were stable against enzymatic attack. However, the diol moiety in the ribose ring of ATP forms a more stable ester with PBA than does the polyol moiety in flexible polymer chain. Therefore, PBA-polyol crosslinking in the PM core will be cleaved by intracellular ATP, which will cause the release of cargo RNA into the cytosol. 

Capelôa et al. synthesized a polypept(o)ide-based copolymer and prepared PIC micelles with small interfering RNAs. These ABC-type polypeptoid-block-polypeptide triblock copolymer consisted of polysarcosine (pSar) block, a poly(S-ethylsulfonyl-L-cystein) (pCys(SO_2_Et)) block, and a poly(L-lysine) block [46]. Specifically, block A has a shielding function, block B acts as a bioreversible and chemically selective crosslinker, and block C can achieve electrostatic binding to siRNA. As displayed in Figure 11C, the PIC micelles can be intrinsically stabilized by introducing a disulfide-crosslinked core using triethyltetramine dicysteine (cTETAc). These PIC micelles effectively transport functional siRNA to Neuro2A and KB cells, but the knockdown efficacy needs improvement.

Aydinlioglu et al. used amphiphilic poly(ethylene oxide)-*b*-poly(L-glutamic acidx-*co*-L-phenylalaniney) and poly(ethylene oxide)-*b*-poly(L-lysine-*co*-L-phenylalaniney) to form siRNA-loaded PICsomes. The good stability of PICsomes, even in high-salinity media, is attributed to the π-π stacking hydrophobic interactions between the Phe residues of the PEO-*b*-poly(amino acids) diblock copolymers [47]. 

### 4.2. PIC Nanoparticles for Gene Delivery

**Figure 12:**
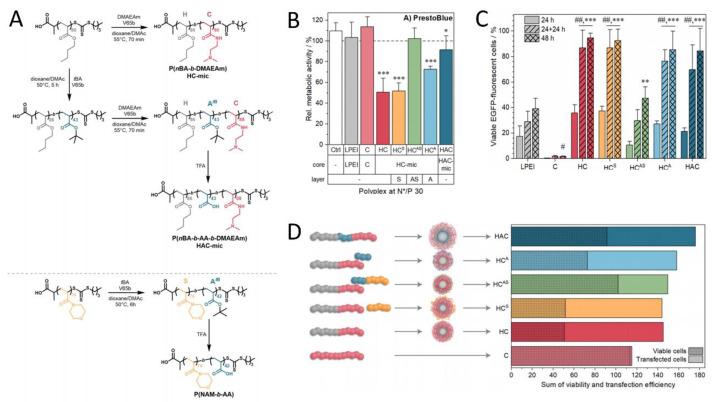
(**A**) Synthesis of block copolymers. (**B**) Toxicity of different polyplexes in HEK293T cells. at N*/P 30 for 24 h. (**C**) Transfection efficiency of PICs in HEK293T cells. Values represent mean ± SD (n = 3). ^#^/^##^ Significant difference to HC-mic at respective time points (*p* < 0.001), */**/*** significant difference to same polymer after 24 h (*p* < 0.05/0.01/0.001) (**D**) Summary of the effects of different polymers on the performance of HC-mic [48].

As a component of PIC nanoparticles, cationic polymers not only increase cytotoxicity but also increase the risk of interaction with serum proteins due to their charged nature, thus limiting their potential applications in the delivery of anionic drugs in vivo. Anionic shielding polymers or hydrophilic polymers have been applied to counteract these effects. Richer et al. [48] synthesized a range of micelle-shielding and micelle-forming polymers via reversible-addition fragmentation chain-transfer polymerization for gene delivery and studied how to avoid the toxicity–efficiency dilemma through composition and assembly. Firstly, cationic micelles (HC-mic) were assembled with diblock copolymer poly[(n-butyl acrylate)-*b*-(2-(dimethyl amino)ethyl acrylamide)](P(nBA-*b*-DMAEAm). The hydrophobic PnBA block enhanced the stability of the PIC micelles carrying DNA. Then, different polymers with shielding functions were incorporated; examples include poly(4-acryloyl morpholine) (PNAM, HC^S^) and poly(acrylic acid) (PAA, HC^A^), which have hydrophilic stealth and negative charge, respectively, or a combination of both was incorporated within a diblock copolymer (P(NAM-*b*-AA), HC^AS^) (Figure 12A). Furthermore, these systems were compared to commercial linear PEI, PDMAEAm, and triblock copolymer micelles (P(nBA-*b*-AA-*b*-DMAEAm), HAC-mic) in terms of transfection efficiency, cytotoxicity, and endosome escape. As shown in Figure 12B, the assembly of HC^S^ and HC-mic exhibited the greatest decrease in cell viability (≈50%) [49]. Adding anionic polymer, HC^A^, or HAC-mic micelles achieved moderate to low toxicity, while HC^AS^ resulted in cytotoxic effects. Then, the transfection efficiency and viability of several micelles were evaluated (Figure 12C). After 48 h, the transfection efficiencies of HC-mic, HAC-mic, and HC^A^ were 95 ± 4%, 85 ± 14%, and 85 ± 18%, respectively. Due to cytotoxicity, HC-mic performed less well than HAC-mic at higher polymer concentrations. The anionic polymer PAA reduced cytotoxicity without influencing the efficiency of the transfection, either as part of the HAC-mic or as part of the HC^A^. These findings prove that the performance of the system depends on the precise molecular arrangement of the polymer blocks (Figure 12D).

Bhowmik et al. prepared soluble micelles using oppositely-charged random copolymers: cationic random copolymers (PCm) consisting of 2-(methacryloyloxy)ethyl phosphorylcholine (MPC; P) with methacroylcholine chloride and anionic random copolymers (PSn) consisting of MPC and potassium 3-(methacryloyloxy) propanesulfonate [50]. These PIC micelles are candidates for delivery of plasmid DNA and oligonucleotides.

**Figure 13:**
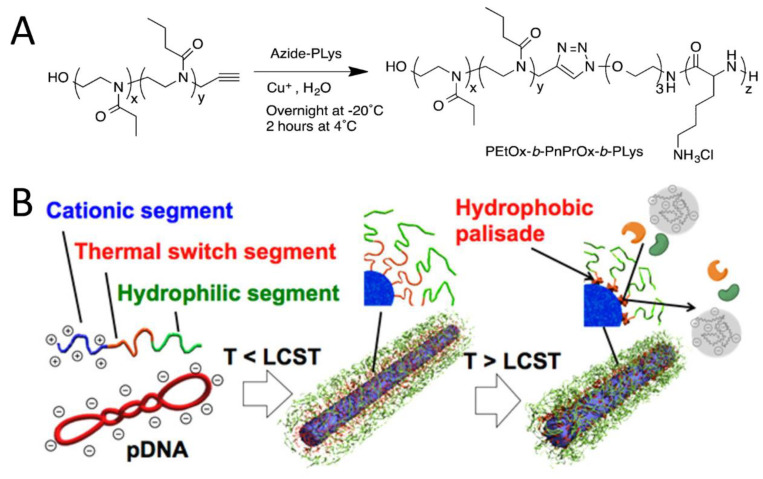
(**A**) Chemical structure of triblock copolymer of PEtOx-*b*-PnPrOx-*b*-Plys. (**B**) Schematic illustration of rod-shaped PIC micelles with spatially regulated hydrophilic-hydrophobic double protective compartments. Image reproduced from reference [51].

Besides spherical micelles and PICsomes, Osada et al. developed interesting cylindrical PIC nanoparticles with thermoswitchable properties and enhanced stability for delivery of plasmid DNA (pDNA) [51]. Using poly(2-ethyl-2-oxazoline) (PEtOx) instead of PEG, triblock copolymers with a thermoresponsive poly(2-n-propyl-2-oxazoline) (PnPrOx) block, PEtOx-*b*-PnPrOx-*b*-PLys, were synthesized (Figure 13A). Mixing pDNA with this copolymer below the lower critical solution temperature (LCST, below 30 °C) of PnPrOx and then incubating the mixture above the LCST to form a hydrophobic palisade of the collapsed PnPrOx segment generated spatially aligned hydrophilic–hydrophobic double-protected rod-shaped PIC nanoparticles (Figure 13B). The rod lengths of the PIC micelles decreased from an average of 135 nm to 90.0 nm after incubation at 37 °C. The hydrophobic palisades were positioned between the pDNA core compartment and the outer shell of micelles and contributed to improvement in the stability of PIC nanoparticles against attacks from both DNase I and chondroitin sulfate, thereby increasing the gene-transfection efficiency. 

## 5. PIC Micelles as an MRI Contrast Agent

**Figure 14:**
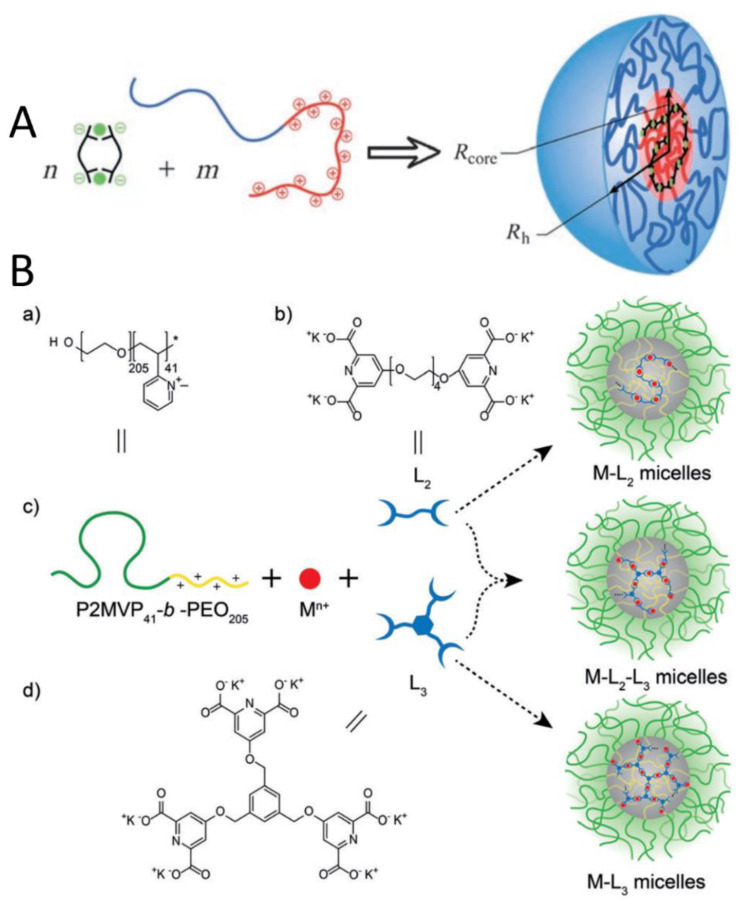
(**A**) Diagram of the formation of complex coacervate core micelles in the mixed system [52]. (**B**) (a) Structure of P2MVP_41_-*b*-PEO_205_. (b) Structure of bis-ligand L_2_. (c) Illustration of the formation of M-L_2_-L_3_ micelles. (d) Structure of tris-ligand L_3_ [53]. Reproduced from [52,53] with permission from Wiley VCH.

Cohen et al. prepared complex coacervate core micelles (C3Ms) from Zn^2+^ ions, 1,11-bis(2,6-dicarboxypyridin-4-yloxy)-3,6,9-trioxaundecane(L_2_EO_4_), and poly(2-vinyl-N-methylpyridinium iodide)-*b*-poly(ethylene oxide) (PMVP_41_-*b*-PEO_205_) [52]. The PIC micelles obtained from a mixture of metal ions (green circles), bisligands, and cationic-neutral diblock copolymer exhibited well-defined, stable, and equilibrated core-shell nanostructures (Figure 14A). Later, this research group developed a triple ligand consisting of three DPA groups fused onto the benzene ring (L_3_) [53]. Combining mixtures of this tris-ligand (L_3_), Mn^II^ ions, bis-ligand (L_2_) with a polycationic-neutral diblock copolymer P2MVP_41_-*b*-PEO_205_, they obtained PIC micelles in which the number of cross-links in the micellar core was regulated simply by varying the L_3_/L_2_ ratio (Figure 14B). Of note, these mixed Mn-L_2_-L_3_ micelles exhibited enhanced stability against salt, excellent biocompatibility, high relaxivity, and strong contrast enhancement in a T_1_-weighted MRI test, which make them very interesting candidates as in vivo MRI contrast agents.

## 6. Conclusions and Outlook

The stability of PIC nanoparticles is the most important factor to be addressed in improving the efficiency of drug delivery. This review summarized the approaches attempted to improve the stability of PIC nanoparticles, such as enhancing the charge density, crosslinking, increasing hydrophobic interactions, adding hydrogen bonding, forming a PIC-based gel, and increasing the rigidity of the PIC component. These methods have demonstrated how chemical or physical approaches can be utilized to improve the stability of PIC nanoparticles and provide the basis for future PIC-based drug-carrier development. It has been demonstrated that enhancing the stability of PIC nanoparticles can prolong circulation in vivo, achieve controlled release of drugs, and increase drug loading. Furthermore, PIC nanoparticles as drug carriers are evolving from their initial use as carriers of gene or protein drugs to uses as carriers of anticancer drugs, peptide antibiotics, and even contrast agents for MRI. 

Although significant research progress has been made, there are not many clinical studies of PIC nanoparticles. One clinical example is [54], which investigated the in vivo theophylline (TH)-release property from TH-loaded PIC tablets, which were prepared by direct compression from a mixture of negatively charged carboxymethyldextran and positively charged [2-(diethylamino)ethyl] dextran as a matrix capable of forming a polyion complex (PIC-tablet). The saliva TH level-time profile revealed that these tablets could deliver sustained drug release. Furthermore, no significant decrease in saliva level-time curves (AUC) was observed for enteric-coated PIC tablets, indicating that PIC tablets had an advantage over conventional hydroxypropylcellulose-based tablets. 

In order to promote the clinical development of PIC nanocarriers, several directions for future research were discussed, as follows. (1) One research direction is employment of the above-mentioned strategies in combination to further improve the stability of PIC. For instance, ionic and hydrogen bonding have been achieved jointly in PIC by using a guanidinium group simultaneously as a hydrogen-bond source and a cation source to bind the carboxyl groups of copolymers [25]. The obtained PICsomes were stable in media with high urea concentrations and under serum incubation, persisting in the blood circulation. (2) Another direction is the combination of organic PICs with inorganic materials to fabricate multifunctional nanocarriers. Kataoka’s group has made progress in a novel siRNA nanocarrier of precisely regulated size constructed from monodisperse unimer PIC and gold nanotemplate [55]. The synergy of the specific function of gold or silver nanoparticles like PTT and antibacterial effect with the drug-delivery function of PIC could be expected. (3) A third direction involves improving the ability to target PIC nanoparticles to the disease site through decoration with antibody fragments [56] or other active targeting groups. (4) A fourth direction is the development of stimuli-responsive PIC micelles for better controlled release of drugs. One mechanism involves introducing ATP-sensitive crosslinkers [44] or ROS-responsive groups into the PIC core. Another is to replace the PEG shell with stimuli-responsive polymers, like temperature-responsive PNIPAM [57]. (5) Various alternatives to PEG have been tried to improve the stealth of nanomaterials, including polyglycerol, hyaluronic acid, poly(2-ethyl-2-oxazoline), ginsenoside [58], DNA [59], and a pluronic copolymer [60]. Nevertheless, most of the reported stealth nanoparticles, including PEGylated PIC systems, showed a rapid drop in blood concentration to half of the administered dose within 1 h post administration, partly due to the increasing prevalence of anti-PEG antibodies in the bloodstream. Therefore, there is enormous room for improvement in the structures of PIC nanoparticles as drug carriers to improve pharmacokinetic behavior and enhance bioavailability. (6) Since most of the peptide, protein and nucleic drugs are negatively charged in physiological conditions, multiple types of drugs could be loaded into the same PIC nanoparticles simultaneously to achieve synergistic therapeutic effects in the future.
